# Precision chest sculpting: 3D-customized silicone implants and computer-aided design assisted reconstruction for pectus excavatum - a single center experience

**DOI:** 10.1016/j.jpra.2026.09.011

**Published:** 2026-09-15

**Authors:** Daniela Geisler, Christoph Müller, Jose R. Matilla, Clemens Aigner, Christine Radtke

**Affiliations:** a University Department of Plastic, Reconstructive and Aesthetic Surgery, Medical University of Vienna, Austria; b University Department of Dermatology, Medical University of Vienna, Vienna, Austria; c University Department of Thoracic Surgery, Medical University of Vienna, Vienna, Austria

**Keywords:** Funnel chest, Chest wall deformities, Congenital deformity, Custom made implant, Thoracic reconstruction

## Abstract

Pectus excavatum correction in adults is often pursued for functional and aesthetic reasons, yet modified Nuss and Ravitch procedures may be invasive and can leave residual anterior chest wall contour deformities. We report a single-center retrospective consecutive case series of aesthetic chest wall correction using patient-specific silicone implants designed with computed tomography (CT)-based three-dimensional (3D) computer-aided design (CAD), with descriptive assessment of postoperative patient-reported and morphological outcomes. Between 2018 and 2024, seven adults underwent CAD-assisted correction at a tertiary referral center (mean age 31.3 ± 11.0 years). Five had previously undergone modified Nuss or Ravitch repair. The primary outcome was postoperative chest-appearance satisfaction, assessed using a six-item questionnaire derived from the modified Nuss questionnaire and scored on a 5-point Likert scale. A mean overall satisfaction score (range 1–5) was calculated. As a secondary outcome, postoperative chest wall morphology was documented using upright 3D surface models (VECTRA WB360) for qualitative illustrative assessment of contour correction. At a mean follow-up of 3.2 ± 1.9 years, patient-reported outcome data were available for five patients and postoperative 3D surface models for four. Mean overall satisfaction was 3.3 ± 0.8 (range 2.3–4.1), with higher satisfaction for chest appearance when clothed than unclothed (4.0 ± 1.0 vs 2.8 ± 1.48). Qualitative 3D assessment suggested restoration of sternal projection and improved chest wall symmetry in all imaged patients. One patient developed postoperative seroma followed by infected hematoma requiring implant explantation. In this retrospective case series, available follow-up data suggested qualitative contour improvement and generally favorable patient-reported satisfaction, but also a non-negligible complication risk. Findings should be interpreted cautiously given the small sample, incomplete follow-up, and descriptive design.

## Introduction

Pectus excavatum is the most common congenital chest wall deformity, defined by posterior displacement of the sternum and adjacent costal cartilages, creating a funnel-shaped anterior chest wall. It affects approximately 1 in 300–400 live births and shows a marked male predominance (approximately 3–4:1).^1^ Pectus excavatum may occur as an isolated anomaly or in association with heritable connective-tissue disease and other genetic conditions, most notably Marfan syndrome, as well as selected monogenic disorders and numeric or structural chromosomal aberrations. The deformity often becomes more pronounced during the pubertal growth spurt and can be accompanied by cardiopulmonary dysfunction and psychosocial burden. Although its etiology remains incompletely understood, current hypotheses implicate abnormal growth and biomechanical remodeling of the costal cartilages and sternum on a genetically susceptible background.^1^^,^^2^ A large proportion of these patients also present with concomitant spine deformity at the time of diagnosis. Such deformities may further complicate surgical correction, particularly at the level of the manubrium.

Clinical presentation ranges from no symptoms to exercise intolerance related to impaired cardiac function and, in some cases chest pain may be present at the time of diagnosis.^3^ The psychosocial burden of the cosmetic deformity frequently predominates, particularly in adolescents, manifesting as body-image concerns, low self-esteem, and social withdrawal.^2^^,^^4^^,^^5^

Severity is most commonly quantified by the Haller Index (HI), calculated on axial CT as the ratio of the maximal internal transverse thoracic diameter to the minimal anteroposterior distance between the posterior sternum and the anterior vertebral body. An HI ≥ 3.5 is widely used to denote a severe defect and is often considered in surgical decision-making.^6^ Concomitant spine deformities can further add to the complexity of surgical correction.

The first report of operative correction was published by Meyer in 1911, who attempted to remove the overgrown rib cartilage by resecting the second and third costal cartilages on the right side only. Because the pectus excavatum deformity remained cosmetically uncorrected, the procedure was considered unsuccessful. One of the earliest techniques still in use was described almost four decades later, in 1949, by Ravitch. This open repair including bilateral resection of the deformed costal cartilages, sternal osteotomy and mobilization, and anterior fixation with supporting bars, and it remained the standard approach for decades, with numerous subsequent modifications.^7^^,^^8^ In 1997, nearly fifty years later, Nuss et al. introduced a minimally invasive repair (MIRPE) using thoracoscopic guidance to place a retrosternal metal bar.^9^^,^^10^ After modified Ravitch or Nuss procedures, the implanted metal bars are typically removed after two to four years.^11^, ^12^, ^13^, ^14^

More recently, treatments targeting cosmetic deformity have gained traction, including autologous fat grafting and implantation of custom-designed silicone prostheses.^15^, ^16^, ^17^, ^18^ Chavoin et al. reported the first large series of patient-specific implants, using three-dimensional (3D) computed tomography (CT) data to design custom prostheses in a series of 401 cases over two decades.^15^ In this approach, CT-based 3D reconstructions are processed using computer-aided design (CAD) software to generate implants tailored to the desired chest wall contour. Patient-specific silicone implants are intended to restore the external anterior chest wall contour and should therefore be regarded as an aesthetic reconstructive option; they do not correct intrathoracic volume reduction or cardiopulmonary compression caused by the underlying deformity.

The aim of this study was to present our single-center experience with CAD-assisted, patient-specific silicone implants for pectus excavatum correction and to provide a descriptive assessment of postoperative aesthetic, patient-reported, and morphological outcomes in a retrospective consecutive case series.

## Materials and methods

### Study design and cohort

A single-center, retrospective consecutive case series was conducted at the University Department of Plastic, Reconstructive and Aesthetic Surgery, Medical University of Vienna. From 2018 to 2024, all patients who underwent correction of pectus excavatum using patient-specific polymerized silicone implants (NuSil MED-4805, Carpinteria, California, USA) manufactured through a 3D CAD workflow (AnatomikModeling, Toulouse, France) were included. The CAD-based manufacturing process has been described in detail by Chavoin et al. and involves acquisition of a CT scan with 1-mm slices, which is then processed by AnatomikModeling for computer modelling and design of the custom implant.^15^

Inclusion criteria were a clinical diagnosis of pectus excavatum with a predominantly aesthetic indication and a patient-expressed desire for external contour correction. Patients selected for silicone implantation were not treated with this approach for correction of cardiopulmonary compression. Exclusion criteria were active infection, uncontrolled cardiopulmonary disease, or prior chest wall irradiation, as these were considered contraindications to silicone implant placement.

The study protocol was approved by the institutional review board and conducted in accordance with the Declaration of Helsinki (EK-1384/2025). The study was reported in accordance with the STROBE (Strengthening the Reporting of Observational Studies in Epidemiology) guidelines. All patients provided written informed consent for study participation.

### Surgical procedure

In all cases, surgery was performed with the patient in the supine position. Preoperative markings and pocket dimensions provided by the manufacturer were used to delineate the planned submuscular pocket (see Fig. 1). Intraoperatively, the patient-specific prototype was used to confirm midline alignment and adjust the pocket borders as needed. Depending on the deformity and on whether concomitant bar removal was performed, either a transverse subxiphoid or a vertical presternal skin incision was used. A submuscular pocket was then created by dissecting down to the sternum and elevating the medial fibers of the pectoralis major muscle together with the anterior fascia of the rectus abdominis muscle. The silicone implant was gently inserted into the pocket and a closed-suction (Redon) drain was placed. The pocket was closed in three layers, and skin closure was achieved with an absorbable intradermal running suture. All patients received single-shot perioperative antibiotic prophylaxis according to institutional standards, and a pressure garment was fitted postoperatively and worn for six to eight weeks.

**Fig. 1:**
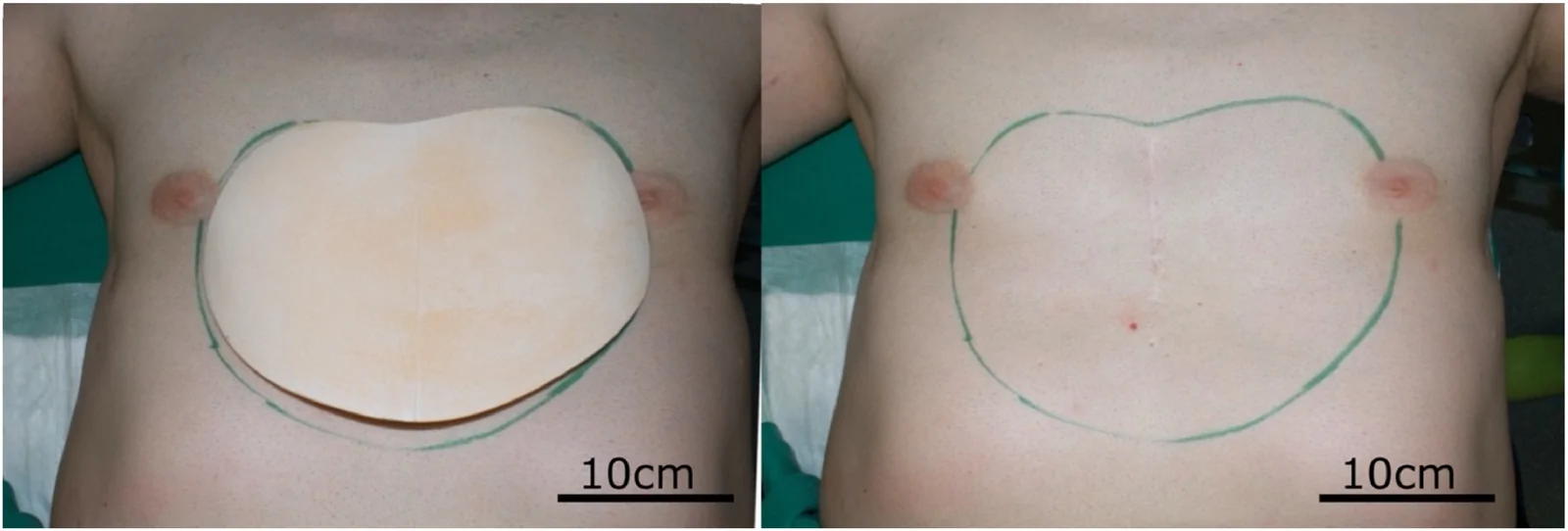
Preoperative markings for patient-specific silicone implant.

The solid implant prototype is used to verify midline alignment on the thorax. Its dimensions guide the preoperative skin markings, which outline the planned submuscular pocket and define the limits of prosthesis placement.

### Outcomes

The primary outcome was patient-reported satisfaction with postoperative chest appearance, evaluated using six items derived from the modified Nuss questionnaire addressing body image, social confidence, and satisfaction with chest appearance.^4^^,^^9^^,^^10^ The questionnaire comprised six items, each rated on a 5-point Likert scale from 1 (very dissatisfied) to 5 (very satisfied). An overall satisfaction score was calculated as the mean of the six items (mean item score; range 1–5), with higher values indicating greater satisfaction. The assessed domains wer:e^1^ satisfaction with chest appearance when clothed,^2^ satisfaction with chest appearance when unclothed,^3^ comfort with removing the shirt in public (e.g., swimming),^4^ confidence in social situations,^5^ satisfaction with the surgical result, and^6^ overall self-image after correction.

As a secondary outcome, postoperative chest wall morphology was documented using 3D surface models generated with the VECTRA WB360 whole-body 3D imaging system (Canfield Scientific). Postoperative surface images were qualitatively compared with the preoperative CT-based virtual reconstruction to illustrate contour correction. Because preoperative CT imaging was acquired in the supine position whereas postoperative VECTRA images were obtained in the standing position, quantitative comparison between modalities was not considered valid.

### Statistical analysis

Given the small sample size, incomplete outcome availability, and the ordinal nature of the Likert-scale questionnaire data, patient-reported outcome (PROM) analyses were restricted to descriptive statistics. Patient characteristics, operative variables, complications, and questionnaire responses are summarized descriptively. The overall satisfaction score is presented as the mean item score (range 1–5), together with item-level results, standard deviations, and ranges. All analyses were performed using R (version 4.5.1; R Foundation for Statistical Computing).

## Results

Between 2018 and 2024, seven patients with pectus excavatum underwent correction with customized silicone implants at the University Department of Plastic, Reconstructive and Aesthetic Surgery, Medical University of Vienna. The cohort comprised seven adults (six men and one transgender man) with a mean age of 31.3 ± 11.0 years (range, 22–51) and mean BMI of 24.5 ± 4.4 kg/m² (range, 20–31). Most patients presented with an asymmetric deformity corresponding to Chin’s morphological classification type 3 (n = 4), whereas three patients had a symmetric depression: two with a shallower symmetric defect extending into the pectoral regions (Chin type 2) and one with a deep symmetric depression centered on the sternum (Chin type 1; see Table 1).^19^

**Table 1:** Patient characteristics (n = 7).

Variable	Value
Age, years (±SD)	31.3 ± 11.0
Sex, male, n (%)	6 (85.7)
BMI, kg/m², mean ± SD	24.5 ± 4.4
Chin type, n	1: 1; 2: 2; 3: 4
Haller index, mean ± SD (previously operated)	3.5 ± 0.8
Haller index, mean ± SD (without prior surgical repair)	4.4 ± 2.4
Prior Nuss repair, n (%)	5 (71.4)
Interval from Nuss to implantation, years, mean ± SD	3.5 ± 1.1

BMI – body mass index, SD – standard deviation.

Among patients with a history of previous pectus repair (n = 5), the interval between the first procedure and implant surgery was 3.5 ± 1.1 years (range, 1.6–4.4). The HI at the time of surgery was 3.5 ± 0.8 (range, 2.8–4.7) in previously operated patients and 4.4 ± 2.4 (range, 2.7–6.1) in patients without prior surgical repair. In four patients, bar removal was performed concomitantly with silicone implantation. The mean operative time for implantation and bar removal was 83.7 ± 41.3 min. The mean length of hospital stay was 12.6 ± 2.6 days. This reflected local postoperative management, as closed-suction drains were routinely maintained until discharge, and in four patients implant placement was combined with bar removal. Early postoperative seroma after drain removal occurred in 1 of 7 patients (14.3%), and the same patient subsequently developed an infected hematoma requiring implant explantation (see Table 2).

**Table 2:** Postoperative results summary (n = 7).

Variable	Value
Concomitant bar removal, n (%)	4 (57.1)
Operative time, min ± SD	83.7 ± 41.3
Postoperative complications, n (%)	1 (14,3%)
Mean length of hospital stay, days	12.6 ± 2.6
Follow-up duration, years, mean ± SD	3.2 ± 1.9
PROM available, n (%) Overall satisfaction score ± SD Satisfaction with chest appearance when clothed ± SD Satisfaction when unclothed ± SD Comfort with removing the shirt in public ± SD Satisfaction with the surgical result ± SD Overall self-image after correction ± SD Confidence in social situations ± SD	5 (71.4) 3.3 ± 0.8 4.0 ± 1.0 2.8 ± 1.48 3.2 ± 1.64 3.4 ± 0.55 3.2 ± 0.45 3.6 ± 0.55
Postoperative 3D surface models available, n (%)	4 (57.1)

PROM – patient reported outcome measures, SD -standard deviation.

Postoperative assessments were performed at a mean follow-up of 3.2 ± 1.9 years (range, 1.3–5.5 years) and were incompletely available: 3D surface models were obtained in four patients (two did not attend follow-up imaging, and one required implant explantation due to infected hematoma). Qualitative assessment of the postoperative 3D surface models showed correction of the anterior depression with restoration of sternal projection and improved chest wall symmetry in all imaged cases (see Fig. 2, Fig. 3). No quantitative 3D comparison metrics were applied because preoperative CT and postoperative surface imaging were obtained in different body positions.

**Fig. 2:**
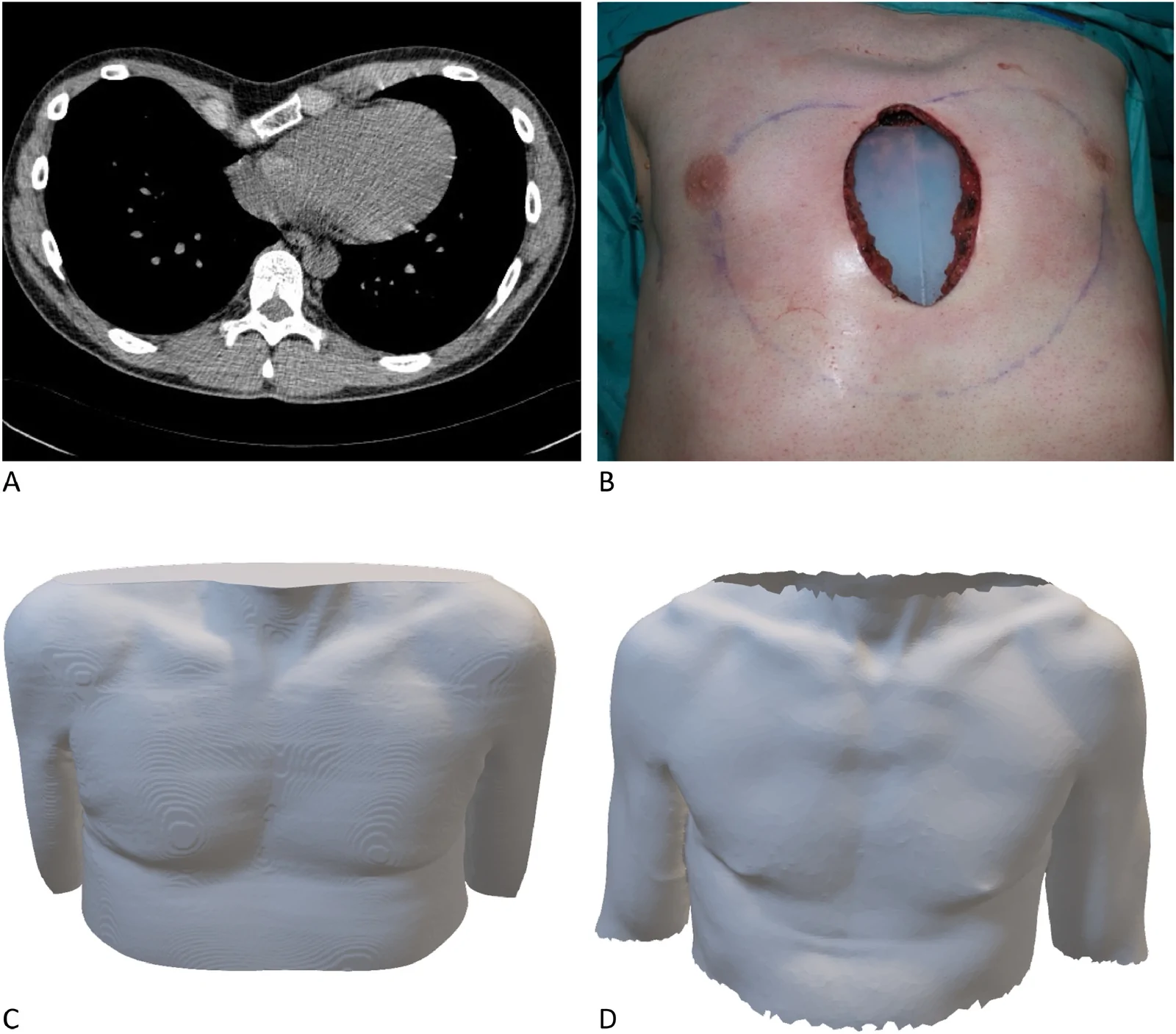
Patient 5 – intraoperative view and imaging of customized implant placement.

(A) Intraoperative view through a vertical presternal incision showing the patient-specific silicone implant seated in the submuscular pocket. (B) Preoperative computed tomography (CT) scan demonstrating the pectus excavatum deformity. (C) Preoperative 3D reconstruction of the thorax used for computer-aided design of the implant. (D) Postoperative 3D VECTRA surface model showing restoration of anterior chest wall contour

**Fig. 3:**
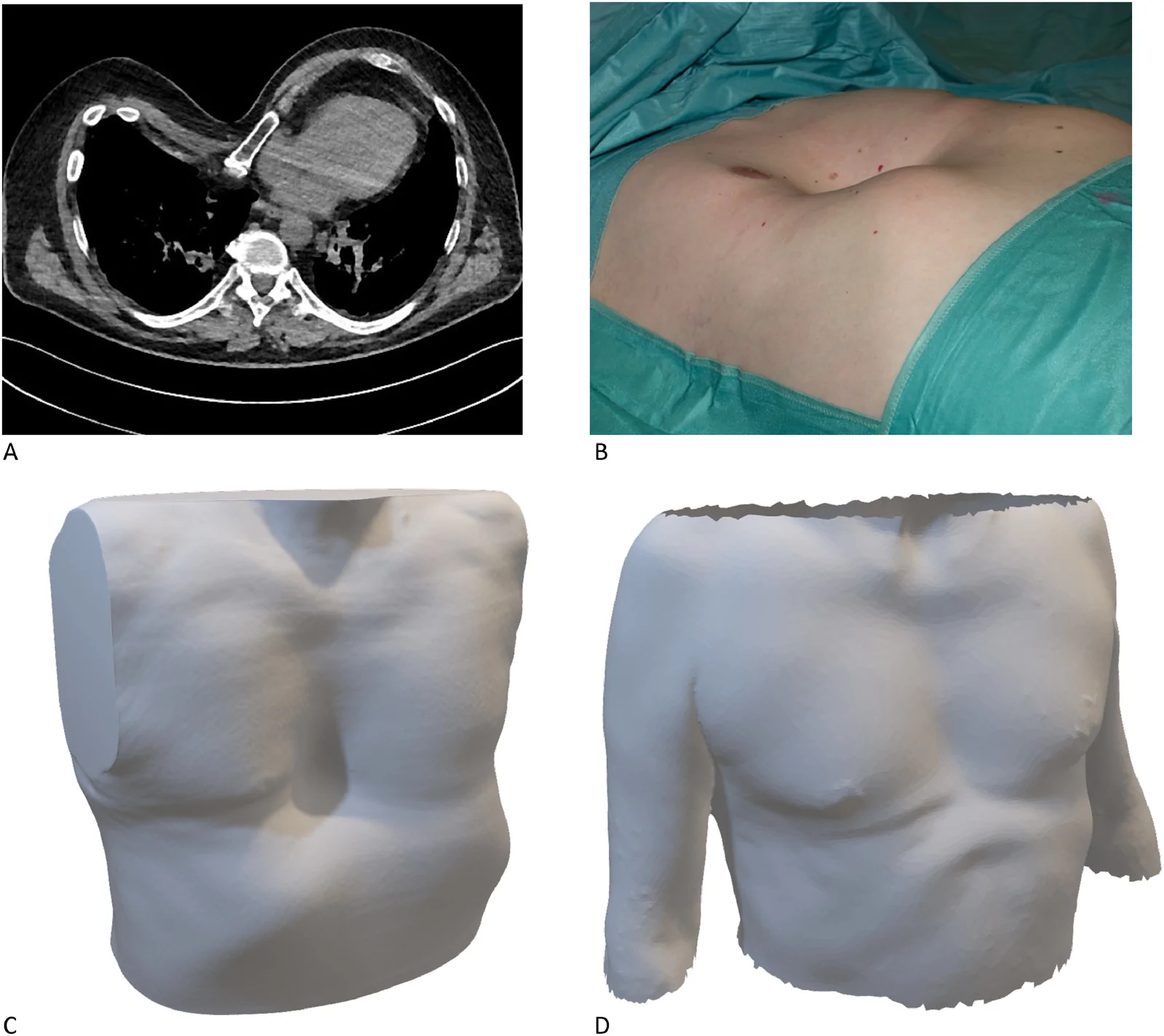
Patient 7 – severe asymmetric pectus excavatum and postoperative correction.

(A) Preoperative clinical view in the operating room from a right inferolateral perspective. (B) Preoperative CT scan demonstrating a Chin type 3 pectus excavatum deformity. (C) Preoperative 3D reconstruction of the thorax. (D) Corresponding postoperative 3D VECTRA surface model illustrating correction of the anterior depression with improved sternal projection and chest wall symmetry.

PROM data were available for five patients at follow-up. The mean overall satisfaction score was 3.3 ± 0.8 (range, 2.3–4.1). Overall scores greater than 3.0 were observed in 3 of 5 patients (60%). Satisfaction with chest appearance was higher when clothed (4.0 ± 1.0; range, 3–5) than when unclothed (2.8 ± 1.48; range, 1–5). Comfort with removing the shirt in public was moderate and variable (3.2 ± 1.64; range, 1–5). Satisfaction with the surgical result was generally favorable (3.4 ± 0.55; range, 3–4), as were ratings for overall self-image after correction (3.2 ± 0.45; range, 3–4) and confidence in social situations (3.6 ± 0.55; range, 3–4). Given the limited sample size and incomplete outcome availability, these findings are descriptive only.

## Discussion

In this small case series of seven adults with predominantly severe pectus excavatum, correction with patient-specific silicone implants was associated with generally favorable aesthetic and patient-reported outcomes among patients with available follow-up data. At a mean follow-up of 3.2 ± 1.9 years, qualitative assessment of postoperative 3D surface models showed restoration of sternal projection and improved chest wall symmetry in all imaged patients.

The management of pectus excavatum has evolved into a multidisciplinary field that integrates corrective techniques from several surgical disciplines.^20^ Within this spectrum, customized silicone implants represent a minimally invasive option primarily aimed at cosmetic restoration and correction in adults with residual deformity. The use of silicone implants for pectus excavatum correction was first described by Murray et al. in 1965.^21^^,^^22^ Since then, the original intraoperative molding technique has largely been replaced by CT-based 3D reconstruction and CAD, enabling precise characterization of the anterior chest wall deformity. Our cohort was characterized by marked deformity severity: overall, 5 of 7 patients (71%) had previously undergone surgical repair and had a HI of ≥3.5 at the time of implantation, with mean HI values of 3.5 ± 0.8 in previously operated patients and 4.4 ± 2.4 in those without prior surgical repair, indicating persistent chest wall depression.^6^^,^^23^ Residual deformity after previous corrective surgery, in combination with predominant cosmetic concerns, therefore constitutes the main indication for this technique in our setting, which is embedded within a multidisciplinary chest wall program involving plastic and thoracic surgeons. In patients with relevant functional limitations, spine deformities, or genetic disorders including Marfan syndrome, open surgical reconstruction remains the preferred option. In contrast, customized implants may be considered when the primary goal is aesthetic restoration of external chest wall contour, especially in localized defects and in postoperative residual deformities after modified Nuss or Ravitch procedures.^24^

Chavoin et al. reported the largest experience with patient-specific silicone implants and emphasized the importance of evaluating patient satisfaction, as the psychological and aesthetic considerations are central in pectus excavatum repair.^15^ In our series, satisfaction and psychosocial outcomes were assessed using items derived from the modified Nuss questionnaire.^25^ Among the five patients with available questionnaire data, the mean overall satisfaction score was 3.3 ± 0.8 on a 1–5 scale, and three patients (60%) reported scores above 3.0. Item-level results showed higher satisfaction with chest appearance when clothed (mean 4.0 ± 1.0) than when unclothed (mean 2.8 ± 1.5), suggesting more favorable patient perception of chest contour when clothed than when unclothed, while some patients remained aware of residual contour irregularities when undressed. Comfort with removing the shirt in public and confidence in social situations were in the mid-to-upper range (means 3.2 and 3.6, respectively), indicating a potential psychosocial benefit in some patients. Lower satisfaction was mainly reported by individuals who described discomfort related to prosthesis awareness, a phenomenon analogous to implant awareness described in other prosthetic procedures and previously noted by Chavoin et al.^15^ These findings underscore the importance of careful preoperative counselling and realistic expectation setting.

Beyond patient-reported outcomes, the safety profile of this technique is an equally important consideration. In our cohort, early postoperative seroma occurred in 1 of 7 patients (14.3%). The same patient developed an infected hematoma requiring explantation; no other major complications or reoperations were observed. These findings underscore that silicone implantation remains subject to the typical risks of foreign-body surgery and requires meticulous hemostasis and careful pocket management. In comparison, Chavoin et al. reported seroma formation in essentially all cases in their series of 401 custom-made implants, although major complications were uncommon. In their protocol, no suction drains were used; instead, compressive dressing was applied for one week and planned seroma aspiration was performed at the first follow-up visit.^15^ Two other studies using custom-made implants based on cast models of the sternal area also reported notable complication rates. In one series of 16 patients, complications occurred in seven patients (44%), with seroma in five patients (31%); two patients required reoperation for implant dislocation secondary to seroma, one required explantation because of persistent seroma, and two additional implants were removed because of pain or discomfort.^18^ Another small series of seven patients reported no immediate complications.^17^ Taken together, these data suggest that patient-specific silicone implants are associated with a non-negligible complication risk that must be carefully balanced against their potential aesthetic benefit, particularly in procedures performed for primarily cosmetic indications. The relatively long mean hospital stay in our cohort reflects local perioperative practice, as closed-suction drains were routinely maintained until discharge and four patients underwent concomitant bar removal in addition to implant placement. It should therefore not be interpreted as representative of silicone implant placement alone.

An additional strength of this case series is the integration of 3D surface imaging into postoperative outcome assessment, which in the imaged patients provided a qualitative illustration of correction of the anterior depression with improved symmetry. To our knowledge, this is the first series to report the use of 3D surface models for postoperative assessment of patient-specific silicone implants in pectus excavatum. An important methodological aspect of our workflow is that preoperative planning was based on CT scans acquired in the supine position, whereas postoperative 3D surface images were obtained with the patient standing upright. This mismatch in positioning may alter the apparent chest wall geometry, as soft-tissue distribution and thoracic configuration change under gravity when moving from lying to standing. Therefore, the postoperative 3D surface images in this study should be interpreted as a qualitative and illustrative tool rather than a basis for formal quantitative comparison with the preoperative CT-based reconstruction. Nevertheless, 3D surface models added a visual dimension to the assessment of contour correction and may support future quantitative analyses of volume restoration and symmetry. This non-invasive tool may also facilitate pre- and postoperative visualization for patients and support shared decision-making, particularly when discussing primarily aesthetic interventions.

This study has several limitations. First, it should be interpreted as a small retrospective single-center case series with descriptive postoperative outcomes assessment rather than a formal effectiveness study. Because PROM data were available for only five patients, patient-reported outcome findings should be interpreted descriptively only; no formal inference regarding effectiveness can be made. In addition, the six-item questionnaire used in this study was derived from the modified Nuss questionnaire and was not separately validated in this reduced form or in adults undergoing aesthetic implant-based correction. Outcome availability was incomplete, with postoperative 3D surface imaging obtained in four patients only, and the absence of a comparator group precludes any causal conclusions regarding treatment effectiveness. Follow-up did not systematically assess cardiopulmonary function or long-term implant-related complications such as capsular contracture, implant displacement, or late infection. Therefore, our data cannot address the functional impact or long-term durability of this approach. Prospective, ideally multicenter studies incorporating standardized PROMs, quantitative 3D surface analyses, and longer-term follow-up on implant behavior are needed to define more clearly the role of patient-specific silicone implants within multidisciplinary chest wall programs.

## Conclusion

In this small retrospective case series, CT-planned patient-specific silicone implants provided an individualized option for aesthetic correction in selected adults with primary or residual pectus excavatum deformities, including patients who declined or were not considered suitable for further major thoracic procedures. Available follow-up data suggested generally favorable patient-reported satisfaction and qualitative improvement in chest wall contour on postoperative 3D surface imaging. These findings also highlight the potential role of 3D surface imaging in future planning and outcome assessment workflows. However, these observations should be interpreted cautiously given the small sample size and incomplete follow-up.

## Declaration of AI and AI-assisted technologies in the writing process

During the preparation of this work, the authors used OpenAI to assist with language editing, stylistic refinement, and improvement of clarity. After using this tool, the authors reviewed and edited the content as needed and take full responsibility for the content of the publication.

## Ethical approval

Ethical approval for this study was obtained from the institutional review board of the Medical University of Vienna (EK-1384/2025). The study was conducted in accordance with the Declaration of Helsinki.

## Funding

None.

## Declaration of competing interest

None declared.
